# Patient and Tumor Factors Influencing Diagnostic Trajectories in Gastrointestinal Stromal Tumor Patients and Their Impact on Health-Related Quality of Life: A Dutch Multicenter Study

**DOI:** 10.1245/s10434-026-19763-2

**Published:** 2026-05-14

**Authors:** Tessa van Amerongen, Emily I. Holthuis, Deborah van de Wal, Dide den Hollander, Ingrid M. E. Desar, Hans Gelderblom, Astrid W. Oosten, Anna K. L. Reyners, Neeltje Steeghs, Joost S. Groen, Winette T. A. Van der Graaf, Olga Husson

**Affiliations:** 1 Department of Medical Oncology, Antoni van Leeuwenhoek Hospital - Netherlands Cancer Institute, Amsterdam, the Netherlands; 2 Department of Medical Oncology, Erasmus MC Cancer Institute, Erasmus University Medical Center, Rotterdam, the Netherlands; 3 Department of Medical Oncology, Radboud University Medical Center, Nijmegen, the Netherlands; 4 Department of Medical Oncology, Leiden University Medical Center, Leiden, the Netherlands; 5 Department of Medical Oncology, University Medical Center Groningen, Groningen, the Netherlands; 6 Department of Medical Oncology, University Medical Center Utrecht, Utrecht, the Netherlands; 7 Patient Platform Sarcomas, Utrecht, the Netherlands; 8 Department of Surgical Oncology, Erasmus MC Cancer Institute, Erasmus University Medical Center, Rotterdam, the Netherlands; 9 Department of Public Health, Erasmus University Medical Center, Rotterdam, the Netherlands

**Keywords:** Gastrointestinal stromal tumor, Diagnostic interval, Diagnostic delay, Health-related quality of life, Patient-reported outcomes

## Abstract

**Background:**

Delays in diagnosis are common in rare cancers, but their frequency and impact in gastrointestinal stromal tumors (GISTs)—one of the most prevalent sarcoma subtypes—are unknown. This study aimed to (1) quantify patient- and diagnostic-related intervals, (2) identify factors influencing interval length, and (3) assess their impact on health-related quality of life (HRQoL).

**Patients and Methods:**

Patients with GIST diagnosed in the Netherlands between 2008 and 2018 completed a questionnaire assessing time to diagnosis, impact of the diagnostic trajectory, and HRQoL. Univariable logistic regression analyses identified associated patient and tumor characteristics.

**Results:**

Among 328 patients (median 5.9 years after diagnosis), 28% reported a patient interval of ≥ 1 month and 14% ≥ 3 months. Diagnostic intervals were longer, with 43% ≥ 1 month and 20% ≥ 3 months. Prolonged diagnostic intervals were more common among female patients and those with small-intestinal GISTs or mid-sized tumors (OR 1.67, 95% CI 1.06–2.64; OR 1.83, 95% CI 1.07–3.16; OR 2.11, 95% CI 1.09–4.14). A total of 20% reported that the diagnostic trajectory negatively impacted their well-being. Actual interval length was not correlated with HRQoL. However, patients perceiving a negative impact had lower HRQoL scores and more often experienced prolonged intervals (68%) than patients reporting no or positive impact (*p* < 0.001). Perceived delay was commonly associated with psychological distress and prolonged physical limitations, often persisting long after diagnosis.

**Conclusions:**

Diagnostic intervals were prolonged in nearly half of patients with GIST, particularly in female patients and those with small-intestinal tumors or mid-sized tumors. Perceived, rather than actual, interval duration was associated with lower HRQoL, underscoring the importance of patient experience during the diagnostic trajectory.

**Supplementary Information:**

The online version contains supplementary material available at 10.1245/s10434-026-19763-2.

Gastrointestinal stromal tumors (GISTs) are rare mesenchymal malignancies originating in the gastrointestinal tract, with an annual incidence of approximately 1–1.5 cases per 100,000 individuals.^1^ Despite their rarity, GISTs represent the most common subtype of soft tissue sarcomas (STS), predominantly located in the stomach (56%) and the small intestine (32%).^2–4^ Clinical presentation is heterogeneous; 18% of cases are asymptomatic.^4,5^ When symptoms occur, they may be acute—such as abdominal pain from obstruction or gastrointestinal bleeding—or nonspecific, including anemia, weakness, weight loss, abdominal distension, ulcer-like complaints, and early satiety.^5–8^

Diagnosis remains challenging due to rarity, nonspecific symptoms, and the need for specialized histopathological and molecular confirmation. At diagnosis, 20–30% of patients present with metastases, most commonly to the liver or peritoneum.^9,10^ Complete surgical resection is the only curative treatment for localized GISTs > 2 cm.^11^ This is often combined with (neo)adjuvant tyrosine kinase inhibitors (TKIs) in high-risk GISTs,^12,13^ as defined by the AFIP-Miettinen criteria.^14^ However, advanced or metastatic GISTs are generally unresectable, and patients often depend on life-long TKI therapy—underscoring the importance of timely diagnosis to enable potentially curative treatment.^15^

The diagnostic trajectory is commonly divided into two intervals: (1) the patient interval, from symptom onset to first healthcare consultation, and (2) the diagnostic interval, from initial consultation to confirmed diagnosis (Fig. 1).^16–18^ Previous studies such as the SURVSARC study and the QUEST study identified prolonged diagnostic intervals and risk factors such as female sex, but excluded patients with GIST.^19,20^ A recent study specifically investigated the prediagnostic general practitioners’ (GPs) pathway of patients with GIST, revealing increased GP consultations starting 4 months before diagnosis,^21^ suggesting possible diagnostic uncertainty. However, the consequences of diagnostic delays remain insufficiently understood.

**Fig. 1:**
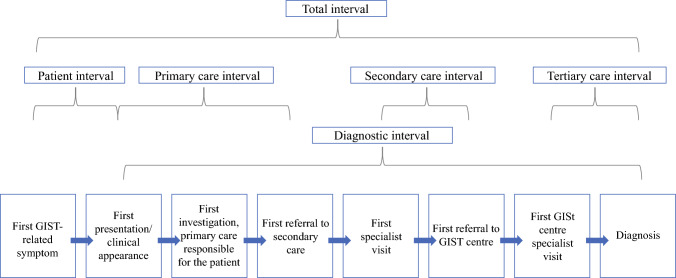
Time intervals used in the questionnaires; in the route from first symptom until diagnosis; total interval: from first GIST-related symptom to diagnosis; patient interval: from the date the patient first noticed a GIST-related symptom until the first presentation to a doctor with this symptom; diagnostic interval: from first presentation to a doctor until diagnosis; primary care interval: from first presentation to a general practitioner until first referral to secondary care (if applicable); secondary care interval: from first appointment in secondary care until referral to tertiary care (specialized GIST center); tertiary care interval: from first appointment at a specialized GIST center until diagnosis; adapted from Olesen et al.^16^ as in Soomers et al.^20^

Concern about prolonged diagnostic intervals is based on the assumption that early diagnosis improves outcomes, including local disease control, overall survival, and health-related quality of life (HRQoL).^17,22^ Nevertheless, data on the length and impact of diagnostic intervals in patients with GIST remain limited. Identifying patient subgroups at increased risk of prolonged diagnostic intervals is crucial for developing strategies to shorten these intervals. Therefore, we conducted a nationwide cross-sectional study in the Netherlands to (1) quantify diagnostic and patient-related intervals, (2) identify patient- and tumor-related factors influencing their duration, and (3) examine the impact of diagnostic trajectories on HRQoL.

## Patients and Methods

### Study Design and Participants

A population-based cross-sectional study (Life with GIST) was conducted among patients with GIST (≥ 18 years) registered in the Netherlands Cancer Registry (NCR). The NCR, which receives newly diagnosed patients from the National Pathology Database (PALGA), was used to select patients diagnosed with GIST (ICD-10-GM codes C15-20, C26, C48, and C80) between January 2008 and December 2018. Data were registered by the Netherlands Comprehensive Cancer Organization (IKNL) and included patient characteristics, tumor staging, and initial treatment. Only patients diagnosed or treated at the five specialized GIST centers described previously were included.^23^ Exclusion criteria included cognitive impairment, current severe illness (as determined by the treating specialist), death, or inability to read Dutch. Ethical approval was obtained from the Radboud University Medical Center ethics committee (2017-3944).

### Data Collection

After informed consent—including linkage to NCR data—patients completed questionnaires online or on paper. Data were collected within the Patient-Reported Outcomes Following Initial Treatment and Long-term Evaluation of Survivorship (PROFILES) registry between September 2020 and June 2021.^24^ Questionnaires were developed by the study group to assess diagnostic intervals and were based on adapted standardized definitions proposed by Olesen et al., as outlined by Soomers et al. (Fig. 1).^16,20^

Intervals were categorized as: < 1 week, 1–2 weeks, 2–4 weeks, 1–3 months, 3–6 months, 6–12 months, and > 12 months. For each interval, patients could also select “I don’t remember” (Fig. S1). Additional response options addressed referral pathways (Fig. S2). The perceived impact of diagnostic interval length on HRQoL was assessed using a single question: “Do you think your HRQoL was influenced by your diagnostic interval length?” Response options included: yes, negatively; yes, positively; or no, followed by an open text field for elaboration.

### Patient and Tumor Characteristics

Patients self-reported sociodemographic characteristics (age, marital status, education). Clinical characteristics (tumor localization, treatment phase, and type) were obtained after linkage with the Dutch GIST Registry (DGR), and comorbidities were assessed using the Self-Administered Comorbidity Questionnaire.^25^ Additional data on gender, socioeconomic status (SES), and missing information were obtained from the NCR. For subgroup analyses, patients were categorized by age at diagnosis as early onset (< 60 years) or late-onset (≥ 60 years) on the basis of the mean age of 60.8 (±10.6) years. SES was categorized as low (≤ 5) or high (≥ 6) on the basis of household income at the postal code level. Patients were also classified at the time of questionnaire completion as receiving curative or palliative treatment.

### Health-Related Quality of Life

HRQoL was measured using the European Organisation for Research and Treatment for Cancer Quality of Life Questionnaire C30 version 3.0 (EORTC QLQ-C30), a 30-item questionnaire covering functional domains (physical, emotional, cognitive, social, and role), global health status, financial impact, and common cancer-related symptoms.^26^ Most items used a 4-point Likert scale (1 = not at all to 4 = very much), while global health items used a 7-point scale (1 = very poor to 7 = excellent). Scores were linearly transformed to a 0–100 scale, with higher scores reflecting better functioning or, for symptoms, greater severity. Analyses focused on global QoL and functional scales. Clinical relevance was determined using the guidelines of Cocks et al. for effect sizes: trivial, small, medium, and large.^27^

### Statistical Analysis

Descriptive statistics summarized categorical variables (frequencies, percentages) and continuous variables (means with standard deviations). Diagnostic intervals were dichotomized into < 1 month and ≥ 1 month, in alignment with previous literature and Dutch SONCOS (Dutch Oncology Collaboration) guidelines,^19,20,28^ which recommend a maximum of 4 weeks from first outpatient visit to histological diagnosis; an additional cutoff of 3 months identified very long intervals. Patients selecting “I don’t remember” were excluded. A subset of patients (*n* = 19) provided inconsistent responses for the secondary and tertiary care intervals; these were treated as missing in the main analysis. Sensitivity analysis recoded interpretable responses, and unclear cases remained missing. Results were compared with the main analysis and showed no material differences. Univariable logistic regression analyses identified factors associated with intervals of ≥ 1 and ≥ 3 months, reported as odds ratios (ORs) with 95% confidence intervals (CIs). Tumor size was first analyzed as a continuous variable; to explore potential nonlinear associations it was subsequently categorized into quartiles on the basis of its distribution in the cohort.

Independent samples *t*-tests compared HRQoL scores across intervals (< 1 versus ≥ 1 month). To examine the association between perceived impact of diagnostic interval length and HRQoL, a one-way analysis of variance (ANOVA) compared global QoL scores across three perception groups (negative, positive, or none), followed by analysis of covariance (ANCOVA), adjusting for gender, time since diagnosis, number of comorbidities, and treatment intent. Significant differences were further explored using Bonferroni post hoc tests. Missing EORTC QLQ-C30 items were handled using mean imputation per EORTC Quality of Life Group guidelines.^29^

A qualitative analysis was performed on responses to an open-ended question completed by patients reporting a negative impact of their diagnostic interval on HRQoL: “Do you believe that your well-being (such as your quality of life) has been affected by the length of your diagnostic trajectory? Yes, negatively, namely:” Responses were independently analyzed by two investigators (T.v.A. and E.I.H.) using inductive coding, followed by axial coding to identify overarching themes. Discrepancies were resolved through discussions, and illustrative quotes were selected to support findings.

All statistical analyses were performed in RStudio (version 2022.07.2) using two-sided tests with a significance threshold set at* p* < 0.05. Missing data were assumed to be missing at random.

## Results

Of 521 invited patients diagnosed with GIST, 328 completed the questionnaire (response rate 63.0%). At diagnosis, the mean patient age was 60.8 (±SD 10.6) years; at questionnaire completion, participants were a mean of 5.9 (±SD 2.8) years post-diagnosis. Most were male (53.0%) and had ≤ 1 comorbidity (55.2%). The majority had high SES (*n* = 178, 54.3%) and reported low-to-intermediate educational levels (*n* = 204, 64.0%). Tumor size had a median of 70.0 mm (range 7.5–300.0 mm). Most patients had GISTs located in the stomach and underwent surgical resection. Of all patients who underwent surgery, 83.3% had planned surgery, 6.7% had planned surgery due to suspicion of a different tumor, and 7.0% underwent emergency surgery; 83.3% of surgeries were performed at centers of expertise. At inclusion, 79.1% of patients were either disease-free or were receiving treatment with curative intent. Demographic and clinical characteristics are summarized in Table 1.

**Table 1:** Sociodemographic and clinical characteristics

	Total (*n* = 328)
Sex, *n* (*%*)	
Male	174 (53.0)
Female	154 (47.0)
Age at diagnosis, m*ean ± SD*	60.8 ± 10.6
Socioeconomic status, *n* (*%*)	
Low	150 (45.7)
High	178 (54.3)
Marital stage, *n* (*%*)	
Married/living with partner	246 (75.7)
Not living with a partner	79 (24.3)
Missing	3
Educational level,^a^ *n* (*%*)	
Low/intermediate	206 (64.0)
High	116 (36.0)
Missing	6
Comorbidity, *n* (*%*)	
None	109 (33.4)
1≥ 2	71 (21.8)
Missing	146 (44.8) 2
Time since diagnosis in years, m*ean ± SD*	5.9 ± 2.8
Location primary GIST, *n* (*%*)	
Stomach	207 (63.1)
Small intestine	79 (24.1)
Rectum	21 (6.4)
Other	21 (6.4)
Primary tumor size in mm, *median* (*range*)	70.0 (7.5–300.0)
Missing, *n*	18
Received TKI at some point, *n (%)*	219 (66.8)
Neoadjuvant	39 (17.8)
Adjuvant	45 (20.5)
Neoadjuvant and adjuvant	42 (19.2)
Palliative	93 (42.5)
Current TKI, *n (%)*	116 (35.4)
Received previous surgery for the GIST, *n* (*%*)	
Yes	300 (91.5)
No	28 (8.5)
Reason for surgery, *n* (*%*)	
Planned operation for GIST	250 (83.3)
Planned operation for other tumor than	20 (6.7)
GIST	21 (7.0)
Emergency operation Missing	9 (3.0)
Location of performed surgery, *n* (*%*)	
Center of expertise	250 (83.3)
Center of referral	43 (14.3)
Missing	7 (2.3)
Phase of treatment according to patient-report, *n* (*%*)	
Declared cured, no follow-up	61 (18.8)
Not receiving active treatment, in follow-up	151 (46.5)
Active treatment with curative intent	45 (13.8)
Treated with palliative intent	67 (20.6)
Palliative intent without treatment	1 (0.3)
Missing	3
Treatment setting, *n (%)*	
Curative setting	257 (79.1)
Palliative setting	68 (20.9)
Missing	3

^a^Low (primary and secondary education), intermediate ((secondary) vocational education) and high (higher vocational education and academic education) educational level

*TKI* tyrosine kinase inhibitor, *SD* standard deviation

### Length of Components of the Intervals

**Fig. 2:**
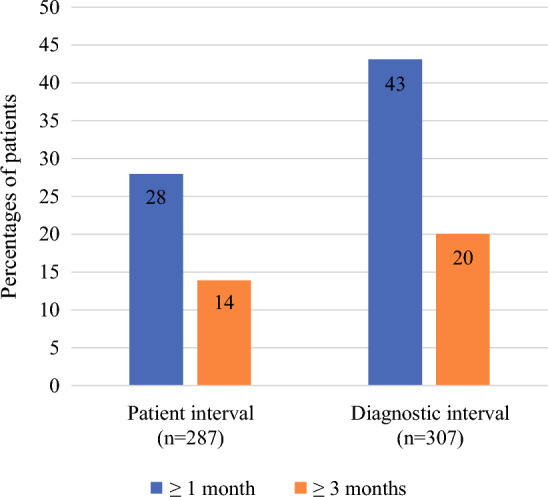
Proportion of patients with patient and diagnostic intervals lasting ≥ 1 month and ≥ 3 months; bar charts showing the proportion of patients reporting a patient interval or diagnostic interval lasting ≥ 1 month (blue) or ≥ 3 months (orange); *Y*-axis truncated at 50% to improve readability; only patients who reported a time interval were included in this analysis

**Fig. 3:**
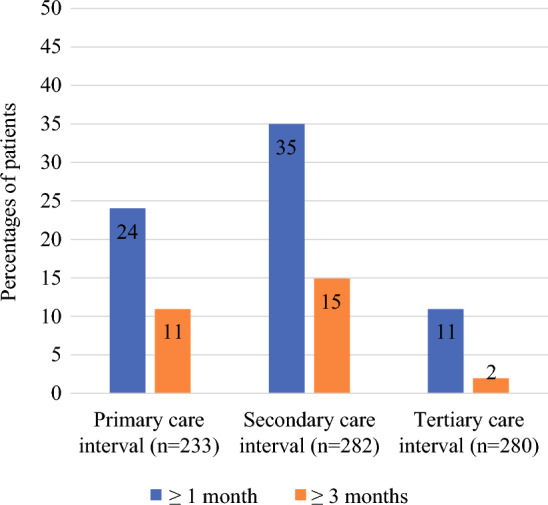
Proportion of patients with primary, secondary, and tertiary care intervals lasting ≥ 1 month and ≥ 3 months; bar charts showing the proportion of patients reporting a primary, secondary, or tertiary care interval lasting ≥ 1 month (blue) or ≥ 3 months (orange); *Y*-axis truncated at 50% to improve readability; only patients who reported a time interval were included in this analysis

The patient-reported intervals for the cohort are presented in Figs. S1 and S2. Data on the patient interval were available for 287 patients, of whom 28% reported ≥ 1 month and 14% ≥ 3 months. A total of 31 patients selected “I do not remember” regarding the duration of their patient interval. Among these, 13 reported that their first medical contact was not with a GP but with another physician (e.g., emergency department), and 9 patients reported no preceding symptoms. The diagnostic interval was reported by 307 patients, with 43% experiencing ≥ 1 month and 20% reported more than 3 months. Within the diagnostic trajectory, patients also responded to questions on specific subintervals (Fig. 2). Data on the primary care interval were available for 233 patients; 72 (31%) patients indicated that they first interacted with a doctor other than their GP about their symptoms. Among those who consulted their GP, 24% reported an interval of ≥ 1 month and 14% exceeded 3 months. In the secondary care interval (*n* = 282), 35% had a duration of ≥1 month and 15% reported durations ≥ 3 months. The tertiary care interval (*n* = 280) was less frequently prolonged, with 11% of patients reporting a duration of ≥ 1 month (Fig. 3).

### Association between Interval Length and Patient and Tumor Characteristics

#### Patient and Diagnostic Interval

Women had longer diagnostic intervals than men; this association was not observed at the ≥ 3-month cutoff. Univariable logistic regression confirmed that women were more likely to have diagnostic intervals ≥ 1 month (OR 1.67, 95% CI 1.06–2.64), as were patients with small-intestinal GISTs (OR 1.83, 95% CI 1.07–3.16). Both associations lost significance at the ≥ 3-month threshold (Tables 2 and S4).

**Table 2:** Univariate logistic regression of association between the (pre)diagnostic intervals and clinical and sociodemographic factors, with a cutoff of 1 month

	Diagnostic interval^a^ ≥ 1 month (versus < 1 month) *N* = 318			Patient interval^b^ ≥ 1 month (versus < 1 month) *N* = 318			Primary care interval^c^ ≥ 1 month (versus < 1 month) *N* = 244			Secondary care interval^d^ ≥ 1 month (versus < 1 month) *N* = 292			Tertiary care interval^e^ ≥ 1 month (versus < 1 month) *N* = 294		
	OR	95% CI	*p*-Value	OR	95% CI	*p*-Value	OR	95% CI	*p*-Value	OR	95% CI	*p*-Value	OR	95% CI	*p*-Value
**Gender**															
Men	1	–	**–**	1	–	–	1	–	–	1	–	–	1	–	–
Women	**1.67**	**1.06–2.64**	**0.028**	1.07	0.63–1.79	0.807	1.21	0.66–2.22	0.540	1.40	0.86–2.29	0.180	1.61	0.77–3.44	0.207
**Age**															
Early onset (< 60)	1	–	–	1	–	–	1	–	–	1	–	–	1	–	–
Late onset (≥ 60)	0.74	0.44–1.24	0.253	0.70	0.44–1.10	0.122	**0.38**	**0.20–0.70**	0.**002**	1.22	0.75–2.02	0.424	0.99	0.47–2.11	0.983
**Socioeconomic status**															
Low	1	–	–	1	–	–	1	–	–	1	–	–	1	–	–
High	0.70	0.44–1.11	0.129	1.22	0.73–2.08	0.451	1.08	0.59–2.00	0.808	0.80	0.49–1.30	0.361	0.78	0.37–1.65	0.518
**Localization**															
Stomach	1	–	–	1	–	–	1	–	–	1	–	**–**	1	–	–
Small intestine	**1.83**	**1.07–3.16**	**0.028**	1.39	0.76–2.53	0.279	1.67	0.82-3.34	0.151	1.62	0.90–2.90	0.105	1.77	0.72–4.28	0.205
Other	1.49	0.75–2.96	0.256	0.93	0.40–1.99	0.855	1.07	0.42-2.50	0.886	1.25	0.59–2.57	0.551	**2.79**	**1.04–7.01**	**0.033**
**Tumor size**^**f**^															
Q1 (< 4.5 cm)	1	–	–	1	–	–	1	–	–	1	–	**–**	1	–	–
Q2 (4.5–7.0 cm)	1.71	0.86–3.43	0.129	1.67	0.69–4.21	0.263	0.61	0.22–1.61	0.325	1.41	0.69–2.88	0.346	0.76	0.26–2.20	0.617
Q3 (7.0–12 cm)	**2.11**	**1.09–4.14**	**0.028**	**2.90**	**1.31–6.85**	**0.011**	0.63	0.26–1.51	0.301	1.55	0.78–3.09	0.214	1.03	0.39–2.72	0.959
Q4 (> 12 cm)	0.86	0.44–1.71	0.672	2.00	0.88–4.80	0.107	0.76	0.33–1.78	0.525	0.84	0.41–1.73	0.642	0.42	0.12–1.31	0.145
**Treatment setting**															
Curative	1	–	–	1	–	–	1	–	–	1	–	–	1	–	–
Palliative	1.16	0.67–1.99	0.606	1.08	0.57–1.98	0.818	**2.44**	**1.26–4.69**	**0.008**	0.88	0.47–1.60	0.674	0.23	0.04–0.81	0.052

Values are *n* (%) or *n*.

^a^Diagnostic interval: from first presentation to a doctor until (histological) diagnosis.

^b^Patient interval: time between start of symptoms and first time the patient talked to a doctor about the symptoms.

^c^Primary care interval: time between first appointment with the GP about the sarcoma-related symptoms and the moment of hospital referral. *Note*: patients who did not consult their GP about sarcoma-related symptoms were excluded from this analysis.

^d^Secondary care interval: time between first appointment in secondary care until referral to a GIST center. *Note*: patients who were directly referred to a GIST center by their GP or who had never visited a GIST center were excluded from this analysis.

^e^Tertiary care interval: time between first appointment in a GIST center and diagnosis. *Note*: patients who had never visited a GIST center were excluded from this analysis.

^f^Tumor size categorized into quartiles on the basis of the distribution within the study cohort.

*Notes*: patients with a missing value for an interval were not taken into account in the analysis; the value could either be missing by randomness or because the patient skipped the question. Bold values correspond to significant differences, *p* < 0.05.

*AYA* adolescents and young adults, *OA* older adults, *OR* odds ratio, *CI* confidence interval

When analyzed continuously, tumor size was not linearly associated with prolonged patient or diagnostic intervals. However, when categorized into quartiles, patients with mid-sized tumors (7.0–12.0 cm) had higher odds of prolonged patient intervals (OR 2.90, 95% CI 1.31–6.85) and diagnostic intervals (OR 2.11, 95% CI 1.09–4.14), whereas the smallest and largest tumors were not associated with prolonged intervals.

#### Primary Care Interval

Patients aged ≥ 60 years had shorter primary care intervals, whereas those treated with palliative intent had longer intervals. Logistic regression confirmed lower odds of a primary care interval ≥ 1 month in patients ≥ 60 years (OR 0.38, 95% CI 0.20–0.70) and higher odds in patients treated with palliative intent (OR 2.44, 95% CI 1.26–4.69). The association with palliative intent remained significant at the ≥ 3-month cutoff (Tables 2 and S4).

#### Secondary Care Interval

No patient or tumor characteristics were associated with the length of the secondary care interval.

#### Tertiary Care Interval

Patients treated with palliative intent were less likely to have prolonged tertiary care intervals (OR 0.23, 95% CI 0.04–0.82), although this was not statistically significant. Patients with GISTs outside the stomach or small intestine had higher odds of prolonged tertiary care intervals (OR 2.79, 95% CI 1.04–7.01). Analyses using a ≥ 3-month cutoff were not feasible due to limited sample size.

#### Health-Related Quality of Life and the Diagnostic Interval

No statistically significant differences in HRQoL scores were observed on the basis of the actual duration of patient or diagnostic intervals (Tables S5 and S6). However, patients’ subjective perceptions of the diagnostic interval’s impact revealed a different association. Among 315 respondents, 59% reported no impact of the diagnostic interval on well-being, 25% reported a negative impact, and 16% reported a positive impact. Participants who reported a negative perceived impact had significantly lower scores on global QoL scale, as well as on emotional, cognitive, and social functioning, compared with those reporting a positive or no impact (Table 3). These differences exceeded thresholds for clinical relevance as defined by Cocks et al. and remained significant after adjusting for gender, time since diagnosis, comorbidity, and treatment intent.^27^

**Table 3:** Mean EORTC QLQ-C30 scores of patients with GIST according to the perceived impact of the diagnostic pathway on well-being

Mean (±SD)	Yes, negatively (*n* = 80)	Yes, positively (*n* = 51)	No impact (*n* = 188)	ANOVA	ANCOVA^c^
Global health status	75.63 (18.69)	*83.67 (12.95)*	*80.98 (16.66)*	**0.0152**^**a**^	**0.005**
Physical functioning	83.58 (16.43)	87.07 (16.52)	87.03 (18.32)	0.318	0.262
Role functioning	81.65 (23.20)	87.76 (20.06)	86.54 (23.71)	0.219	0.178
Emotional functioning^d^	81.12 (23.06)	*93.37 (10.76)*	*91.58 (12.90)*	**< 0.001**^**ab**^	**< 0.001**^**a,b**^
Cognitive functioning	81.86 (20.18)	*85.03 (19.62)*	*89.74 (16.11)*	**0.003**^**b**^	**0.003**^**a**^
Social functioning	84.60 (24.72)	***95.58 (11.17)***	*91.39 (18.35)*	**0.004**^**a,b**^	**0.003**^**b**^

The values in bold indicate a statically significant *p*-value, < 0.05

^a,b^Corresponds to statistically significant Bonferroni post hoc analysis (*p* < 0.05) for (a) negative impact versus positive impact and for (b) negative impact versus no impact.

^c^Analysis of covariance, corrected for gender, time since diagnosis, comorbidity, and treatment setting.

^d^The clinical relevance of the emotional functioning score was determined according to the role functioning interpretation guidelines.

*Notes*: Values in italic correspond to small clinical relevant differences as determined by Cocks et al. and value in bold italic correspond to medium clinical relevant differences as determined by Cocks et al.

*ANOVA* analysis of variance, *ANCOVA* analysis of covariance

**Fig. 4:**
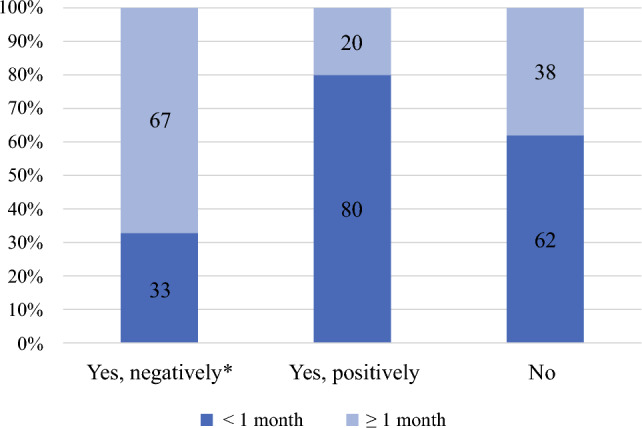
Perceived impact of the diagnostic trajectory on well-being, stratified by interval duration (< 1 month versus ≥ 1 month); *corresponds to statistically significant Bonferroni post hoc analysis (*p* < 0.001), for “yes, negatively” versus “yes, positively” and “yes, negatively” versus “no impact”

Although the actual diagnostic interval duration was not independently associated with HRQoL, participants who perceived a negative impact were significantly more likely to have experienced a prolonged diagnostic interval (≥ 1 month in 68%) than those reporting a positive (20%) or no impact (38%) (Fig. 4). These associations remained significant in the post hoc analysis.

### Qualitative Analysis: Perceived Negative Impact of Diagnostic Interval Length on Well-Being

Four key themes emerged from the qualitative analysis: (1) psychological distress, (2) physical inability, (3) influence on treatment and/or prognosis, and (4) influence of healthcare factors. Among patients who commented on why their well-being was influenced negatively by their diagnostic pathway (*n* = 76), 39% attributed this to psychological distress. This distress was primarily related to uncertainty, ongoing stress, and fear surrounding the diagnostic process. In their own words:

- “Unease caused by uncertainty,” male, 55 years old, 5 years since diagnosis
- “Unnecessarily prolonged tension”, male, 81 years old, 11 years since diagnosis

Physical inability was reported by 33% (*n* = 25), often due to longer symptom duration or permanent symptoms resulting from delayed treatment. Of these, 72% had a diagnostic interval length ≥ 1 month, and 48% ≥ 3 months. For example:

- “Experienced severe symptoms for a long time without knowing the cause. Received incorrect medication and experienced difficulties at work,” female, 62 years old, 5 years since diagnosis
- “To this day, I have limitations that are definitely related to my diagnostic pathway,” female, 60 years old, 6 years since diagnosis

The theme influence on treatment and/or prognosis was reported by 15% of patients. These individuals believed that earlier diagnosis could have resulted in less extensive treatment or reduced the risk of metastases. Illustrative quotes include:

- “Yes, because then the resection would have been complete, and the tumor would not have been adhered to my pancreas,” male, 66 years ldo, 4 years since diagnosis
- “If the GIST had been diagnosed earlier, a less extensive surgery would have been needed to remove the smaller tumor; my recovery would have been better and faster. Moreover, there would have been a lower chance of the metastases I have now,” female, 68 years old, 7 years since diagnosis

Finally, influence of healthcare factors encompassed comments about misdiagnosis and delayed referral. Of these participants, 80% had a diagnostic interval length ≥ 1 month, and 50% ≥ 3 months. For instance:

- “Because the diagnosis could not be established at the regional hospital,” male, 77 years old, 10 years since diagnosis
- “Because they did not consider GIST and focused on various other causes, the diagnosis took a very long time,” female, 72 years old, 5 years since diagnosis

## Discussion

This is the first nationwide study to examine (pre)diagnostic intervals in patients with GIST, identify factors associated with prolonged intervals, and explore their relationship with HRQoL. Our main findings indicate that a proportion of patients experienced prolonged patient and diagnostic intervals. Although the actual duration of intervals was not associated with HRQoL, patients perceiving a negative impact of the diagnostic trajectory on their well-being reported significantly lower HRQoL scores across multiple domains. Importantly, these findings suggest that the way patients experience and interpret their diagnostic trajectory may be more strongly associated with HRQoL than the absolute duration of the interval itself.

Among patients who recalled their patient interval, 28% reported an interval of ≥ 1 month, and 14% ≥ 3 months. Diagnostic intervals were longer, with 43% exceeding 1 month and 20% ≥ 3 months. These prolonged intervals likely reflect GIST’s rarity, nonspecific symptoms, limited awareness among patients and clinicians, and limited availability of expertise in nontertiary centers.

Women, patients with small-intestinal GISTs, and those with mid-sized tumors (7–12 cm) were more likely to experience diagnostic intervals of ≥ 1 month (ORs 1.7, 1.8, and 2.1, respectively). Similar findings in sarcomas and other cancers suggest that sociocultural factors and healthcare biases may contribute to longer pathways despite women’s generally proactive health-seeking behavior.^19,20,30^ For small-intestinal GISTs, prolonged intervals may reflect more subtle symptomatology and limited visualization during standard investigations; a Dutch small-intestinal GIST cohort found anemia was the most frequent symptom (44%).^31^ Gastroscopy and colonoscopy, commonly used for anemia, do not visualize most of the small intestine, thus lesions may be missed, and sequential testing can prolong diagnosis.

The finding that mid-sized tumors were most prone to prolonged diagnostic intervals suggests a nonlinear relationship between tumor size and the diagnostic trajectory. One possible explanation is that mid-sized tumors may produce less specific or less alarming symptoms than very large tumors, while very small tumors may more often be detected incidentally. Mid-sized tumors were also associated with prolonged patient intervals (OR 2.9), which may reflect similar symptom patterns influencing patients’ help-seeking behavior.

Age was not associated with the patient or diagnostic interval. Although longer intervals might be expected in younger patients due to lower suspicion of malignancy, shorter intervals can occur when presentation is acute; a Dutch study reported higher emergency surgery rates in young adults (≤ 40 years).^32^ Patients > 60 years were less likely to have prolonged primary care intervals (OR 0.4), possibly reflecting more proactive diagnostic workups. Patients treated with palliative intent had significantly longer primary care intervals (OR 2.4), which may reflect complex presentations delaying diagnosis and leading to advanced disease. These findings align with reports of multiple GP consultations before GIST diagnosis,^21^ suggesting symptoms that do not immediately raise suspicion of malignancy.

Inefficiencies within the healthcare system also contribute to diagnostic delays. Prior sarcoma research has identified delays due to incorrect initial diagnoses, inefficient investigations, absent coordinating clinicians, and prolonged referrals—factors likely relevant to GIST given its rarity and diagnostic complexity.^33^ In our cohort, the secondary care interval was a key contributor to the diagnostic interval, which may reflect low initial clinical suspicion or delayed recognition of the need for referral at nonspecialized centers. In the Netherlands, most patients first presented to nonspecialized centers (87%) and referral to specialized care was low (36.5%), particularly for lower-risk cases. This is important, because confirming GIST and initiating treatment requires specialized histopathology and molecular analysis,^14^ which are significantly more consistently performed in reference centers (96.9% versus 87.6%).^34^ Notably, about one-third of localized GIST surgeries occur outside reference centers—often before definitive diagnosis due to incidental findings, procedures for other indications, or emergencies.^34^ In our cohort, 14% of surgeries were performed in nonspecialized hospitals, nearly half for emergency or unrelated reasons. These patterns illustrate that in GIST, treatment may occasionally precede diagnosis, complicating interpretations of diagnostic intervals and referral pathways. By contrast, tertiary care intervals were rarely prolonged, indicating efficient processes once patients reach specialized centers.

Although actual interval length was not associated with HRQoL scores, patients’ perceptions of the diagnostic process had a significant impact. Patients who believed that the length of their diagnostic interval had negatively affected their well-being reported substantially lower scores on global QoL, as well as on emotional, cognitive, and social functioning.^27,35^ Notably, 65% of these patients also experienced prolonged intervals, suggesting that subjective experiences often reflect objective reality. Coping strategies, known predictors of HRQoL in other cancers, may further influence these perceptions.^36–38^

Our qualitative analysis supports the association between perceived diagnostic delay and lower HRQoL. Among patients who perceived a negative impact of their diagnostic pathway, psychological distress was most commonly reported and often centered on prolonged uncertainty, frustration, and fear. In addition, many patients described physical limitations resulting from untreated symptoms or delayed care, some of which had long-term consequences for daily functioning or work. Others believed that earlier diagnosis might have reduced treatment intensity or improved prognosis, highlighting the emotional weight of timely diagnosis. Delays were often attributed to healthcare factors—such as misattributed symptoms or inefficiencies—fostering frustration, and a sense of being failed by the system. These experiences often leave a lasting psychological imprint, explaining why subjective experience of delay may effect HRQoL more than objective interval length. Similar findings have been reported in other cancers, where fast-track referrals improve satisfaction and reduce long-term concerns.^39–42^ These findings underscore that not only diagnostic timeliness, but also patients’ subjective experiences during the diagnostic trajectory, are important determinants of long-term HRQoL. Clear communication regarding expected diagnostic timelines and reassurance about oncologic implications, when appropriate, may help reduce long-term psychosocial distress.

This study has several strengths. It is the first to examine GIST diagnostic trajectories and their association with HRQoL. Despite the rarity of GIST, our relatively large sample size (*n* = 328) was broadly representative in tumor location, sex, and age, supporting generalizability. Limitations include potential survivorship bias, as mostly long-term survivors with favorable prognoses were included, and reliance on patient-reported data, which may introduce recall bias. However, patients rarely reported being unable to remember certain interval durations, and cancer diagnosis are generally well remembered.^43^ Another important consideration is the use of the EORTC QLQ-C30, which, although widely validated, may not fully capture the nuanced impact of diagnostic delays. In addition, findings reflect the Dutch healthcare system, where GPs act as gatekeepers; delays may differ in systems with direct specialist access. Future research could minimize these limitations through prospective data collection, such as brief questionnaires about their patient interval, experienced symptoms, and concerns completed by patients and GPs at diagnosis. Thereby, clinically meaningful thresholds should be defined for delay and evaluate their impact on outcomes. Finally, earlier specialist involvement in suspected GIST cases, awareness of sex-related differences, and structured feedback between specialists and GPs could enhance diagnostic timeliness and equity. Addressing the long-term consequences of diagnostic experiences and reinforcing communication—especially particularly management—remain essential to improving quality of life in GIST.

## Conclusions

This nationwide study shows that nearly half of patients with GIST experienced diagnostic intervals of ≥ 1 month, with 20% reporting ≥ 3 months. Prolonged diagnostic intervals particularly affected women, patients with small-intestinal GIST and those with mid-sized tumors (7–12 cm). The secondary care interval substantially contributed to total diagnostic delay, highlighting the importance of timely recognition and referral from nonspecialized centers. Although interval length was not associated with HRQoL, patients perceiving their diagnostic trajectory negatively reported significantly lower HRQoL scores—even years after diagnosis—and often had objectively prolonged intervals. Qualitative findings revealed lasting psychological and physical limitations tied to these experiences. These results underscore the need for diagnostic pathways that integrate clear expectation management and psychosocial support, while providing a foundation for future research to define meaningful delay thresholds. Ultimately, a more patient-centered diagnostic approach could improve long-term care quality in GIST.

## Supplementary Information

Below is the link to the electronic supplementary material.

Supplementary file 1 (DOCX 121 KB)

## Data Availability

Data are available on reasonable request from the corresponding author.
