# Supplementary material for: Patient and Tumor Factors Influencing Diagnostic Trajectories in Gastrointestinal Stromal Tumor Patients and Their Impact on Health-Related Quality of Life: A Dutch Multicenter Study
Source: Ann Surg Oncol. 2026 May 14;33(8):7331–42. doi: 10.1245/s10434-026-19763-2 (PMC13337877; doi:10.1245/s10434-026-19763-2)
Supplement: Supplementary file 1 — (DOCX 121 KB) [file 10434_2026_19763_MOESM1_ESM.docx]

# Patient and Tumor Factors Influencing Diagnostic Trajectories in Gastrointestinal Stromal Tumor Patients and Their Impact on Health-Related Quality of Life: A Dutch Multicenter Study

**Authors**

Tessa van Amerongen^1,2^, Emily I. Holthuis^1,2^, Deborah van de Wal^2^, Dide den Hollander^4^, Ingrid M. E. Desar^3^, Hans Gelderblom^4^, Astrid W. Oosten^2^, Anna K. L. Reyners^5^, Neeltje Steeghs^1,6^, Joost S. Groen^7^, Winette T.A. Van der Graaf^1,2^, Olga Husson^1,8.9^

**Affiliations**

^1^Department of Medical Oncology, Antoni van Leeuwenhoek Hospital - Netherlands Cancer Institute, Amsterdam, The Netherlands

^2^Department of Medical Oncology, Erasmus MC Cancer Institute, Erasmus University Medical Center, Rotterdam, The Netherlands

^3^Department of Medical Oncology, Radboud University Medical Center, Nijmegen, The Netherlands

^4^Department of Medical Oncology, Leiden University Medical Center, Leiden, The Netherlands

^5^Department of Medical Oncology, University Medical Center Groningen, Groningen, The Netherlands

^6^Department of Medical Oncology, University Medical Center Utrecht, Utrecht, The Netherlands

^7^Patient Platform Sarcomas, Utrecht, the Netherlands

^8^Department of Surgical Oncology, Erasmus MC Cancer Institute, Erasmus University Medical Center, Rotterdam, The Netherlands

^9^Department of Public Health, Erasmus University Medical Center, Rotterdam, The Netherlands

**Corresponding author:** Dr. O. Husson, Associate Professor, [o.husson@erasmusmc.nl](mailto:o.husson@erasmusmc.nl), Dr. Molewaterplein 40, 3015 GD Rotterdam, the Netherlands. @OlgaHusson Orchid ID: 0000-0002-1387-8686

**Supplementary Materials - Index**

| **Supplementary Figures and Tables** |  |
| --- | --- |
| Figure S1. Patient-reported lengths of the patient and diagnostic interval | *page 3* |
| Figure S2. Patient-reported lengths of the diagnostic intervals | *page 4* |
| Table S1. Patient and diagnostic intervals, stratified by patient and clinical characteristics, with a cut-off of 1 month. | *page 5* |
| Table S2. Patient and diagnostic intervals, stratified by patient and clinical characteristics, with a cut-off of 3 months. | *page 6* |
| Table S3. Univariate logistic regression of association between the (pre-)diagnostic intervals and clinical and sociodemographic factors, with a cut-off of 3 months. | *page 7* |
| Table S4. Mean EORTC QLQ-C30 scores of GIST patients according to diagnostic interval. | *page 8* |
| Table S5. Mean EORTC QLQ-C30 scores of GIST patients according to patient interval. | *page 8* |
| **References** | *page 9* |
|  |  |

Figure S1. Patient-reported lengths of the patient and diagnostic interval

Figure S2. Patient-reported lengths of the diagnostic intervals

Table S1. Patient and diagnostic intervals, stratified by patient and clinical characteristics, with a cut-off of 1 month.

|  | **Diagnostic interval**^a^  **≥1 month (vs. <1 month)**  **N = 318** | | | **Patient interval**^b^  **≥1 month (vs. <1 month)**  **N = 318** | | | **Primary care interval**^c^  **≥1 month (vs. <1 month)**  **N = 244** | | | **Secondary care interval**^d^  **≥1 month (vs. <1 month)**  **N = 292** | | | **Tertiary care interval**^e^  **≥1 month (vs. <1 month)**  **N = 294** | | |
| --- | --- | --- | --- | --- | --- | --- | --- | --- | --- | --- | --- | --- | --- | --- | --- |
|  | **<1 month** | **≥1 month** | **IDR** | **<1 month** | **≥1 month** | **IDR** | **<1 month** | **≥1 month** | **IDR** | **<1 month** | **≥1 month** | **IDR** | **<1 month** | **≥1 month** | **IDR** |
| **Gender**  Men  Women | 103 (63.2)  73 (50.7) | 60 (36.8)  71 (49.3) | 9  2 | 112 (72.7)  95 (71.4) | 42 (27.3)  38 (28.6) | 18  13 | 99 (78.0)  79 (74.5) | 28 (22.0)  27 (25.5) | 4  7 | 104 (68.4)  79 (60.8) | 48 (31.6)  51 (39.2) | 6  4 | 138 (90.8)  110 (85.9) | 14 (9.2)  18 (14.1) | 7  7 |
| **Age at diagnosis**  Early onset (≤60)  Late onset (>60) | 73 (52.5)  103 (61.3) | 66 (47.5)  65 (38.7) | 4  7 | 88 (68.8)  119 (74.8) | 40 (31.2)  40 (25.2) | 16  15 | 71 (67.0)  107 (84.3) | 35 (33.0)  20 (15.7) | 4  7 | 83 (67.5)  100 (62.9) | 40 (32.5)  59 (37.1) | 3  7 | 108 (88.5)  140 (88.6) | 14 (11.5)  18 (11.4) | 7  7 |
| **Socio-economic status**  Low  High | 72 (52.6)  104 (61.2) | 65 (47.5)  66 (38.8) | 6  5 | 93 (74.4)  114 (70.4) | 32 (25.6)  48 (29.6) | 20  11 | 81 (77.1)  97 (75.8) | 24 (22.9)  31 (24.2) | 5  6 | 82 (62.1)  101 (67.3) | 50 (37.9)  49 (32.7) | 2  8 | 109 (87.2)  139 (89.7) | 16 (12.8)  16 (10.3) | 7  7 |
| **Localization**  Stomach  Small intestine  Other | 120 (62.2)  35 (47.3)  21 (52.5) | 73 (37.8)  39 (52.7)  19 (47.5) | 8  2  1 | 131 (73.6)  46 (66.7)  30 (75.0) | 47 (26.4)  23 (33.3)  10 (25.0) | 22  8  1 | 112 (78.3)  38 (69.1)  28 (77.8) | 30 (21.7)  17 (30.1)  8 (22.2) | 6  4  1 | 122 (68.2)  37 (56.9)  24 (63.2) | 57 (31.8)  28 (43.1)  14 (36.8) | 5  3  2 | 162 (91.5)  55 (85.9)  31 (79.5) | 15 (8.5)  9 (14.1)  8 (20.5) | 8  5  1 |
| **Tumor size^f^**  Q1 (<4.5cm)  Q2 (4.5-7.0cm)  Q3 (7.0-12cm)  Q4 (>12 cm)  Missing^g^ | 44 (63.8)  33 (50.8)  35 (45.5)  53 (67.1)  11 (64.7) | 25 (36.2)  32 (49.2)  42 (54.5)  26 (32.9)  6 (35.3) | 1  3  5  1  1 | 49 (83.1)  44 (74.6)  49 (62.8)  54 (71.1)  11 (73.3) | 10 (16.9)  15 (25.4)  29 (37.2)  22 (28.9)  4 (26.7) | 10  9  4  5  3 | 34 (70.8)  32 (80.0)  50 (79.4)  51 (76.1)  11 (73.3) | 14 (29.2)  8 (20.0)  13 (20.6)  16 (23.9)  4 (26.7) | 1  5  1  4  0 | 42 (65.6)  38 (57.6)  42 (55.3)  52 (69.3)  16 (94.1) | 22 (34.4)  28 (42.4)  34 (44.7)  23 (30.7)  1 (5.9) | 1  3  3  3  0 | 52 (85.2)  53 (88.3)  62 (84.9)  68 (93.2)  16 (100) | 9 (14.8)  7 (11.7)  11 (15.1)  5 (6.8)  0 (0) | 1  3  4  4  2 |
| **Treatment intent**  Curative  Palliative | 140 (58.1)  36 (54.5) | 101 (41.9)  30 (45.5) | 9  2 | 163 (72.4)  44 (71.0) | 62 (27.6)  18 (29.0) | 26  5 | 142 (80.7)  36 (63.2) | 34 (19.3)  21 (36.8) | 8  3 | 144 (64.3)  39 (67.2) | 80 (35.7)  19 (32.8) | 8  2 | 193 (86.5)  55 (96.5) | 30 (13.5)  2 (3.5) | 9  5 |

Values are n (%) or n. AYA, adolescents and young adults; OA, older adults; IDR, I don’t remember; M, metastasis; NA, not applicable.

^a^Diagnostic interval: from first presentation to a doctor until diagnosis.

^b^Patient interval: time between start of sarcoma-related symptoms and first time the patient talked to a doctor about the symptoms. Note: patients who did not consult their GP about sarcoma-related symptoms were excluded from this analysis.

^c^Primary care interval: time between first appointment with the GP about the sarcoma-related symptoms and the moment of hospital referral. Note: patients who did not consult their GP about sarcoma-related symptoms were excluded from this analysis.

^d^Secondary care interval: time between first appointment in secondary care until referral to a GIST centre. Note: patients who were directly referred to a GIST center by their GP or who had never visited a GIST center were excluded from this analysis.

^e^Tertiary care interval: time between first appointment in a GIST centre and diagnosis. Note: patients who had never visited a GIST center were excluded from this analysis.

^f^Tumor size categorized into quartiles based on the distribution within the study cohort.

^g^Patients with missing tumor size were not included in the statistical analyses.

Note I: patients with a missing value for an interval were not taking into account in the analysis, the value could either be missing by randomness or because the patient skipped the question.

Note II: grey shades correspond to significant differences, p<0.05. Statistical tests were performed using Pearson’s Chi-squared test, except when expected cell counts were <5, in which case Fisher’s Exact Test was used.

|  | **Diagnostic interval**^a^  **≥3 month (vs. <3 month)**  **N = 318** | | | **Patient interval**^b^  **≥3 month (vs. <3 month)**  **N = 318** | | | **Primary care interval**^c^  **≥3 month (vs. <3 month)**  **N = 244** | | | **Secondary care interval**^d^  **≥3 month (vs. <3 month)**  **N = 292** | | | **Tertiary care interval**^e^  **≥3 month (vs. <3 month)**  **N = 294** | | |
| --- | --- | --- | --- | --- | --- | --- | --- | --- | --- | --- | --- | --- | --- | --- | --- |
|  | **<3 month** | **≥3 month** | **IDR** | **<3 month** | **≥3 month** | **NS** | **<3 month** | **≥3 month** | **IDR** | **<3 month** | **≥3 month** | **IDR** | **<3 month** | **≥3 month** | **IDR** |
| **Gender**  Men  Women | 136 (83.4)  111 (77.1) | 27 (16.6)  33 (22.9) | 9  2 | 133 (86.4)  115 (86.5) | 21 (13.6)  18 (13.5) | 18  13 | 116 (91.3)  92 (86.8) | 11 (8.7)  14 (13.2) | 4  7 | 133 (87.5)  108 (83.1) | 19 (12.5)  22 (16.9) | 6  4 | 151 (99.3)  123 (96.1) | 1 (0.7)  5 (3.9) | 7  7 |
| **Age at diagnosis**  Early onset (<60)  Late onset (≥60) | 107 (77.0)  140 (83.3) | 32 (23.0)  28 (16.7) | 4  7 | 106 (82.8)  142 (89.3) | 22 (17.2)  17 (10.7) | 16  15 | 90 (84.9)  188 (95.4) | 16 (15.1)  9 (4.6) | 4  7 | 107 (87.0)  134 (84.3) | 16 (13.0)  25 (15.7) | 3  7 | 120 (98.4)  154 (97.5) | 2 (1.6)  4 (2.5) | 7  7 |
| **Socio-economic status**  Low  High | 110 (80.3)  137 (80.6) | 27 (19.7)  33 (19.4) | 6  5 | 110 (88.0)  138 (85.2) | 15 (12.0)  24 (14.8) | 20  11 | 96 (91.4)  112 (87.5) | 9 (8.6)  16 (12.5) | 5  6 | 110 (83.3)  131 (87.3) | 22 (16.7)  19 (12.7) | 2  8 | 120 (96.0)  154 (99.4) | 5 (4.0)  1 (0.6) | 7  7 |
| **Localization**  Stomach  Small intestine  Other | 160 (82.9)  56 (75.7)  31 (77.5) | 33 (17.1)  18 (24.3)  9 (22.5) | 8  2  1 | 158 (88.8)  58 (84.1)  32 (80.0) | 20 (11.2)  11 (15.9)  8 (20.0) | 22  8  1 | 127 (89.4)  50 (90.9)  31 (86.1) | 15 (10.6)  5 (9.1)  5 (13.9) | 6  4  1 | 158 (88.3)  54 (83.1)  29 (76.3) | 21 (11.7)  11 (16.9)  9 (23.7) | 5  3  2 | 175 (98.9)  62 (96.9)  37 (94.9) | 2 (1.1)  2 (3.1)  2 (5.1) | 8  5  1 |
| **Tumor size^f^**  Q1 (<4.5cm)  Q2 (4.5-7.0cm)  Q3 (7.0-12cm)  Q4 (>12 cm)  Missing^g^ | 57 (82.6)  50 (76.9)  56 (72.7)  70 (88.6)  14 (82.4) | 12 (17.4)  15 (23.1)  21 (27.3)  9 (11.4)  3 (17.6) | 1  3  5  1  1 | 54 (91.5)  55 (93.2)  62 (79.5)  64 (84.2)  13 (86.7) | 5 (91.5)  4 (93.2)  16 (79.5)  12 (84.2)  2 (86.7) | 10  9  4  5  3 | 42 (87.5)  38 (95.0)  56 (88.9)  60 (89.6)  12 (80.0) | 6 (12.5)  2 (5.0)  7 (11.1)  7 (10.4)  3 (20.0) | 1  5  1  4  0 | 55 (85.9)  55 (83.3)  58 (76.3)  69 (92.0)  17 (100) | 9 (14.1)  11 (16.7)  18 (23.7)  6 (8.0)  0 (0) | 1  3  3  3  0 | 57 (98.3)  58 (96.7)  70 (95.9)  73 (100)  16 (100) | 1 (1.7)  2 (3.3)  3 (4.1)  0 (0)  0 (0) | 1  3  4  4  2 |
| **Treatment intent**  Curative  Palliative | 197 (81.7)  50 (75.8) | 44 (18.3)  16 (24.2) | 10  1 | 193 (85.8)  55 (88.7) | 32 (14.2)  7 (11.3) | 26  5 | 164 (93.2)  44 (77.2) | 12 (6.8)  13 (22.8) | 8  3 | 188 (83.9)  53 (91.4) | 36 (16.1)  5 (8.6) | 8  2 | 217 (97.3)  57 (100) | 6 (2.7)  0 (0) | 9  5 |

Table S2. Patient and diagnostic intervals, stratified by patient and clinical characteristics, with a cut-off of 3 months.

AYA, adolescents and young adults; OA, older adults; IDR, I don’t remember; M, metastasis; NS, no symptoms; NA, not applicable.

^a^Diagnostic interval: from first presentation to a doctor until diagnosis.

^b^Patient interval: time between start of symptoms and first time the patient talked to a doctor about the symptoms.

^c^Primary care interval: time between first appointment with the GP about the sarcoma-related symptoms and the moment of hospital referral. Note: patients who did not consult their GP about sarcoma-related symptoms were excluded from this analysis.

^d^Secondary care interval: time between first appointment in secondary care until referral to a GIST centre. Note: patients who were directly referred to a GIST center by their GP or who had never visited a GIST center were excluded from this analysis.

^e^Tertiary care interval: time between first appointment in a GIST centre and diagnosis. Note: patients who had never visited a GIST center were excluded from this analysis.

^f f^Tumor size categorized into quartiles based on the distribution within the study cohort.

^g^Patients with missing tumor size were not included in the statistical analyses.

Note I: patients with a missing value for an interval were not taking into account in the analysis, the value could either be missing by randomness or because the patient skipped the question.

Note II: bold values correspond to significant differences, p<0.05.

Table S3. Univariate logistic regression of association between the (pre-)diagnostic intervals and clinical and sociodemographic factors, with a cut-off of 3 months.

|  | **Diagnostic interval**^a^  **≥3 month (vs. <3 month)**  **N = 318** | | | **Patient interval**^b^  **≥3 month (vs. <3 month)**  **N = 318** | | | **Primary care interval**^c^  **≥3 month (vs. <3 month)**  **N = 244** | | | **Secondary care interval**^d^  **≥3 month (vs. <3 month)**  **N = 292** | | | **Tertiary care interval**^e^  **≥3 month (vs. <3 month)**  **N = 294** | | |
| --- | --- | --- | --- | --- | --- | --- | --- | --- | --- | --- | --- | --- | --- | --- | --- |
|  | **OR** | **95% CI** | **P** | **OR** | **95% CI** | **P** | **OR** | **95% CI** | **P** | **OR** | **95% CI** | **P** | **OR** | **95% CI** | **P** |
| **Gender**  Men  Women | 1  1.50 | -  0.85-2.66 | **-**  .163 | 1  1.07 | -  0.50-1.95 | -  .979 | 1  1.60 | -  0.70-3.78 | -  .267 | 1  1.43 | -  0.73-2.79 | -  .295 | 1  *NA* | -  *NA* | -  *NA* |
| **Age**  Early onset (<60)  Late onset (≥60) | 1  0.67 | -  0.38-1.18 | -  .164 | 1  0.58 | -  0.29-1.14 | -  .113 | 1  0.43 | -  0.17-1.00 | -  .054 | 1  1.25 | -  0.64-2.50 | -  .522 | 1  *NA* | -  *NA* | -  *NA* |
| **Socio-economic status**  Low  High | 1  0.98 | -  0.56-1.74 | -  .948 | 1  1.28 | -  0.64-2.60 | -  .491 | 1  1.52 | -  0.66-3.75 | -  .338 | 1  0.73 | -  0.37-1.41 | -  .343 | 1  *NA* | -  *NA* | -  *NA* |
| **Localization**  Stomach  Small intestine  Other | 1  1.56  1.41 | -  0.80-2.96  0.59-3.14 | -  .181  .420 | 1  1.50  1.98 | -  0.66-3.27  0.76-4.74 | -  .319  .140 | 1  0.84  1.37 | -  0.26-2.32  0.42-3.83 | -  .759  .574 | 1  1.53  2.33 | -  0.67-3.33  0.94-5.49 | -  .291  .058 | 1  *NA*  *NA* | -  *NA*  *NA* | -  *NA*  *NA* |
| **Tumor size^f^**  Q1 (<4.5cm)  Q2 (4.5-7.0cm)  Q3 (7.0-12cm)  Q4 (>12 cm) | 1  1.43  1.78  0.61 | -  0.61-3.38  0.81-4.06  0.23-1.54 | -  .413  .157  .300 | 1  0.79  2.79  2.03 | -  0.19-3.12  1.02-8.98  0.70-6.69 | -  .729  .060  .210 | 1  0.37  0.88  0.82 | -  0.05-1.71  0.27-2.90  0.25-2.70 | -  .238  .822  .732 | 1  1.22  1.90  0.53 | -  0.47-3.26  0.80-4.76  0.17-1.56 | **-**  .681  .155  .256 | 1  *NA*  *NA*  *NA* | -  *NA*  *NA*  *NA* | -  *NA*  *NA*  *NA* |
| **Treatment setting**  Curative  Palliative | 1  1.43 | -  0.73-2.71 | -  .279 | 1  0.77 | -  0.29-1.74 | -  .552 | 1  **4.04** | -  **1.72-9.59** | -  .**001** | 1  0.49 | -  0.16-1.21 | -  .158 | 1  *NA* | -  *NA* | -  *NA* |

AYA, adolescents and young adults; OA, older adults; OR, odds ratio; CI, confidence interval; P, P-value; NA., not applicable.

*a*Unreliable OR due to a small sample size, see table 5.

^a^Diagnostic interval: from first presentation to a doctor until diagnosis.

^b^Patient interval: time between start of symptoms and first time the patient talked to a doctor about the symptoms.

^c^Primary care interval: time between first appointment with the GP about the sarcoma-related symptoms and the moment of hospital referral. Note: patients who did not consult their GP about sarcoma-related symptoms were excluded from this analysis.

^d^Secondary care interval: time between first appointment in secondary care until referral to a GIST centre. Note: patients who were directly referred to a GIST center by their GP or who had never visited a GIST center were excluded from this analysis.

^e^Tertiary care interval: time between first appointment in a GIST centre and diagnosis. Note: patients who had never visited a GIST center were excluded from this analysis.

^f f^Tumor size categorized into quartiles based on the distribution within the study cohort.

Note I: patients with a missing value for an interval were not taking into account in the analysis, the value could either be missing by randomness or because the patient skipped the question.

Note II: bold values correspond to significant differences, p<0.05.

Table S4. Mean EORTC QLQ-C30 scores of GIST patients according to diagnostic interval

|  | Total  (n=307) | ≤ 1 month  (n=176) | >1 month  (n=131) | *p*-value (CI) |
| --- | --- | --- | --- | --- |
| Global health status | 79.89 | 80.59 | 79.68 | .639 (-2.90, 4.72) |
| Physical functioning | 85.87 | 87.09 | 85.06 | .320 (-1.98, 6.03) |
| Role functioning | 85.28 | 86.63 | 84.51 | .426 (-3.12, 7.36) |
| Emotional functioning | 89.21 | 89.83 | 88.61 | .533 (-2.62, 5.06) |
| Cognitive functioning | 86.81 | 86.34 | 87.37 | .629 (-5.24, 3.17) |
| Social functioning | 90.24 | 91.67 | 89.06 | .242 (-1.77, 6.98) |

Table S5. Mean EORTC QLQ-C30 scores of GIST patients according to patient interval

|  | Total  (n=287) | ≤ 1 month (n=207) | >1 month  (n=80) | *p*-value (CI) |
| --- | --- | --- | --- | --- |
| Global health status | 79.88 | 80.54 | 78.14 | .290 (-2.07, 6.88) |
| Physical functioning | 85.91 | 86.18 | 85.19 | .678 (-3.70, 5.67) |
| Role functioning | 85.28 | 84.80 | 84.42 | .902 (-5.71, 6.47) |
| Emotional functioning | 89.21 | 89.51 | 87.99 | .480 (-2.73, 5.78) |
| Cognitive functioning | 86.81 | 87.56 | 84.20 | .182 (-1.60, 8.32) |
| Social functioning | 90.24 | 91.06 | 86.58 | .106 (-0.96, 9.91) |

## References

1. Stiller CA, Trama A, Serraino D, et al. Descriptive epidemiology of sarcomas in Europe: report from the RARECARE project. *Eur J Cancer*. Feb 2013;49(3):684-95. doi:10.1016/j.ejca.2012.09.011

2. Søreide K, Sandvik OM, Søreide JA, Giljaca V, Jureckova A, Bulusu VR. Global epidemiology of gastrointestinal stromal tumours (GIST): A systematic review of population-based cohort studies. *Cancer Epidemiol*. Feb 2016;40:39-46. doi:10.1016/j.canep.2015.10.031

3. Board WCoTE. *Soft Tissue and Bone Tumours*. International Agency for Research on Cancer; 2020.

4. Parab TM, DeRogatis MJ, Boaz AM, et al. Gastrointestinal stromal tumors: a comprehensive review. *J Gastrointest Oncol*. Feb 2019;10(1):144-154. doi:10.21037/jgo.2018.08.20

5. Menge F, Jakob J, Kasper B, Smakic A, Gaiser T, Hohenberger P. Clinical Presentation of Gastrointestinal Stromal Tumors. *Visc Med*. Oct 2018;34(5):335-340. doi:10.1159/000494303

6. Rammohan A, Sathyanesan J, Rajendran K, et al. A gist of gastrointestinal stromal tumors: A review. *World J Gastrointest Oncol*. Jun 15 2013;5(6):102-12. doi:10.4251/wjgo.v5.i6.102

7. Joensuu H, Hohenberger P, Corless CL. Gastrointestinal stromal tumour. *Lancet*. Sep 14 2013;382(9896):973-83. doi:10.1016/s0140-6736(13)60106-3

8. Marcella C, Shi RH, Sarwar S. Clinical Overview of GIST and Its Latest Management by Endoscopic Resection in Upper GI: A Literature Review. *Gastroenterol Res Pract*. 2018;2018:6864256. doi:10.1155/2018/6864256

9. van der Graaf WTA, Tielen R, Bonenkamp JJ, Lemmens V, Verhoeven RHA, de Wilt JHW. Nationwide trends in the incidence and outcome of patients with gastrointestinal stromal tumour in the imatinib era. *Br J Surg*. Jul 2018;105(8):1020-1027. doi:10.1002/bjs.10809

10. Schaefer IM, DeMatteo RP, Serrano C. The GIST of Advances in Treatment of Advanced Gastrointestinal Stromal Tumor. *Am Soc Clin Oncol Educ Book*. Apr 2022;42:1-15. doi:10.1200/edbk_351231

11. Bauer S, George S, von Mehren M, Heinrich MC. Early and Next-Generation KIT/PDGFRA Kinase Inhibitors and the Future of Treatment for Advanced Gastrointestinal Stromal Tumor. *Front Oncol*. 2021;11:672500. doi:10.3389/fonc.2021.672500

12. Huang WK, Wu CE, Wang SY, et al. Systemic Therapy for Gastrointestinal Stromal Tumor: Current Standards and Emerging Challenges. *Curr Treat Options Oncol*. Sep 2022;23(9):1303-1319. doi:10.1007/s11864-022-00996-8

13. Casali PG, Blay JY, Abecassis N, et al. Gastrointestinal stromal tumours: ESMO-EURACAN-GENTURIS Clinical Practice Guidelines for diagnosis, treatment and follow-up. *Ann Oncol*. Jan 2022;33(1):20-33. doi:10.1016/j.annonc.2021.09.005

14. Miettinen M, Lasota J. Gastrointestinal stromal tumors: review on morphology, molecular pathology, prognosis, and differential diagnosis. *Arch Pathol Lab Med*. Oct 2006;130(10):1466-78. doi:10.5858/2006-130-1466-gstrom

15. Nikfarjam M, Kimchi E, Shereef S, et al. Surgical outcomes of patients with gastrointestinal stromal tumors in the era of targeted drug therapy. *J Gastrointest Surg*. Nov 2008;12(11):2023-31. doi:10.1007/s11605-008-0561-4

16. Olesen F, Hansen RP, Vedsted P. Delay in diagnosis: the experience in Denmark. *Br J Cancer*. Dec 3 2009;101 Suppl 2(Suppl 2):S5-8. doi:10.1038/sj.bjc.6605383

17. Weller D, Vedsted P, Rubin G, et al. The Aarhus statement: improving design and reporting of studies on early cancer diagnosis. *Br J Cancer*. Mar 27 2012;106(7):1262-7. doi:10.1038/bjc.2012.68

18. Soomers V, Husson O, Young R, Desar I, Van der Graaf W. The sarcoma diagnostic interval: a systematic review on length, contributing factors and patient outcomes. *ESMO Open*. Feb 2020;5(1)doi:10.1136/esmoopen-2019-000592

19. Patient and tumour factors influencing length of sarcoma diagnostic trajectory intervals: first results from the international QUEST study. *Br J Surg*. Mar 4 2025;112(3)doi:10.1093/bjs/znaf021

20. Soomers V, Husson O, Desar IME, et al. Patient and diagnostic intervals of survivors of sarcoma: Results from the SURVSARC study. *Cancer*. Dec 15 2020;126(24):5283-5292. doi:10.1002/cncr.33181

21. Holthuis EI, Slijkhuis V, van der Graaf WTA, et al. The Prediagnostic General Practitioners' Pathway of Gastrointestinal Stromal Tumor Patients: A Real-World Data Study. *Cancers (Basel)*. Apr 22 2025;17(9)doi:10.3390/cancers17091391

22. Neal RD, Tharmanathan P, France B, et al. Is increased time to diagnosis and treatment in symptomatic cancer associated with poorer outcomes? Systematic review. *Br J Cancer*. Mar 31 2015;112 Suppl 1(Suppl 1):S92-107. doi:10.1038/bjc.2015.48

23. van de Wal D, den Hollander D, Desar IME, et al. Financial difficulties experienced by patients with gastrointestinal stromal tumours (GIST) in the Netherlands: data from a cross-sectional multicentre study. *Support Care Cancer*. Apr 10 2024;32(5):279. doi:10.1007/s00520-024-08451-0

24. van de Poll-Franse LV, Horevoorts N, van Eenbergen M, et al. The Patient Reported Outcomes Following Initial treatment and Long term Evaluation of Survivorship registry: scope, rationale and design of an infrastructure for the study of physical and psychosocial outcomes in cancer survivorship cohorts. *Eur J Cancer*. Sep 2011;47(14):2188-94. doi:10.1016/j.ejca.2011.04.034

25. Sangha O, Stucki G, Liang MH, Fossel AH, Katz JN. The Self-Administered Comorbidity Questionnaire: a new method to assess comorbidity for clinical and health services research. *Arthritis Rheum*. Apr 15 2003;49(2):156-63. doi:10.1002/art.10993

26. Aaronson NK, Ahmedzai S, Bergman B, et al. The European Organization for Research and Treatment of Cancer QLQ-C30: a quality-of-life instrument for use in international clinical trials in oncology. *J Natl Cancer Inst*. Mar 3 1993;85(5):365-76. doi:10.1093/jnci/85.5.365

27. Cocks K, King MT, Velikova G, Martyn St-James M, Fayers PM, Brown JM. Evidence-based guidelines for determination of sample size and interpretation of the European Organisation for the Research and Treatment of Cancer Quality of Life Questionnaire Core 30. *J Clin Oncol*. Jan 1 2011;29(1):89-96. doi:10.1200/jco.2010.28.0107

28. SONCOS. Multi-disciplinary standardisation of oncology care in the Netherlands. Accessed January, 2025. <https://demedischspecialist.nl/sites/default/files/2023-06/soncos_normeringsrapport_11_english_version.pdf>

29. Fayers P, Aaronson NK, Bjordal K, Grønvold M, Curran D, Bottomley A. *EORTC QLQ-C30 scoring manual*. 3rd ed. European Organisation for research and treatment of cancer; 2001.

30. Din NU, Ukoumunne OC, Rubin G, et al. Age and Gender Variations in Cancer Diagnostic Intervals in 15 Cancers: Analysis of Data from the UK Clinical Practice Research Datalink. *PLoS One*. 2015;10(5):e0127717. doi:10.1371/journal.pone.0127717

31. Boonstra PA, Steeghs N, Farag S, et al. Surgical and medical management of small bowel gastrointestinal stromal tumors: A report of the Dutch GIST registry. *Eur J Surg Oncol*. Mar 2019;45(3):410-415. doi:10.1016/j.ejso.2018.09.013

32. NS IJ, Drabbe C, den Hollander D, et al. Gastrointestinal Stromal Tumours (GIST) in Young Adult (18-40 Years) Patients: A Report from the Dutch GIST Registry. *Cancers (Basel)*. Mar 20 2020;12(3)doi:10.3390/cancers12030730

33. Soomers V, van der Graaf WTA, Zaidi S, et al. The route to diagnosis of sarcoma patients: Results from an interview study in the Netherlands and the United Kingdom. *PLoS One*. 2020;15(12):e0243439. doi:10.1371/journal.pone.0243439

34. Roets E, Ijzerman NS, Ho VKY, et al. Referral patterns of GIST patients: data from a nationwide study. *Acta Oncol*. Feb 14 2024;63:28-34. doi:10.2340/1651-226x.2024.23722

35. Soomers V, Desar IME, van de Poll-Franse LV, et al. The Perceived Impact of Length of the Diagnostic Pathway Is Associated with Health-Related Quality of Life of Sarcoma Survivors: Results from the Dutch Nationwide SURVSARC Study. *Cancers (Basel)*. Jul 28 2020;12(8)doi:10.3390/cancers12082088

36. Chirico A, Lucidi F, Merluzzi T, et al. A meta-analytic review of the relationship of cancer coping self-efficacy with distress and quality of life. *Oncotarget*. May 30 2017;8(22):36800-36811. doi:10.18632/oncotarget.15758

37. Pan CJ, Liu HC, Liang SY, Liu CY, Wu WW, Cheng SF. Resilience and Coping Strategies Influencing the Quality of Life in Patients With Brain Tumor. *Clin Nurs Res*. Jan 2019;28(1):107-124. doi:10.1177/1054773817714562

38. Jarkovský J, Skřivanová K, Benešová K, et al. Predictors of quality of life in Czech female breast cancer survivors following treatment with special interest to coping strategies. *Vnitr Lek*. Summer 2017;63(6):389-396. Prediktory kvality života u českých pacientek po léčbě karcinomu prsu se zaměřením na dovednosti zvládání zátěže.

39. Dahl TL, Vedsted P, Jensen H. The effect of standardised cancer pathways on Danish cancer patients' dissatisfaction with waiting time. *Dan Med J*. Jan 2017;64(1)

40. Pham TM, Gomez-Cano M, Salika T, Jardel D, Abel GA, Lyratzopoulos G. Diagnostic route is associated with care satisfaction independently of tumour stage: Evidence from linked English Cancer Patient Experience Survey and cancer registration data. *Cancer Epidemiol*. Aug 2019;61:70-78. doi:10.1016/j.canep.2019.04.011

41. Salika T, Abel GA, Mendonca SC, et al. Associations between diagnostic pathways and care experience in colorectal cancer: evidence from patient-reported data. *Frontline Gastroenterol*. Jul 2018;9(3):241-248. doi:10.1136/flgastro-2017-100926

42. Sandager M, Jensen H, Lipczak H, Sperling CD, Vedsted P. Cancer patients' experiences with urgent referrals to cancer patient pathways. *Eur J Cancer Care (Engl)*. Jan 2019;28(1):e12927. doi:10.1111/ecc.12927
